# Association of LR treatment response category with outcome of patients with hepatocellular carcinoma on explant pathology

**DOI:** 10.1007/s00261-025-04811-4

**Published:** 2025-01-25

**Authors:** Aman Khurana, Nathan Chai, Amanda Gibson, Joseph Owen, Ahmed Sobieh, Gregory Hawk, James Lee

**Affiliations:** 1 University of California, San Diego, USA; 2 University of Kentucky, Lexington, USA

**Keywords:** Hepatocellular carcinoma, LI-RADS, Liver transplanation, Disease free survival, Milan criteria, Locoregional therapy, MAFLD, MASH

## Abstract

**Objectives:**

Liver transplant (LT) is an effective treatment for hepatocellular carcinoma (HCC) in appropriately selected patients. Locoregional therapy (LRT) is often performed to extend a patient’s eligibility for LT. Imaging has a modest sensitivity of approximately 40–77% for detecting pathologically viable HCC in post-LRT patients. The impact on overall survival (OS) and disease-free survival (DFS) is unclear. We hypothesize that Liver Imaging Reporting & Data Systems Treatment Response (LI-RADS TR) category is equivalently correlated with long-term survival and overall disease-free progression when compared to explant pathology findings. We additionally hypothesize that neoadjuvant LRT can improve OS and DFS in LT patients initially within MC.

**Methods:**

Patients found to have HCC on explant between January 2005 and December 2021 were included. A total of 167 patients were divided into treatment (any pre-LT LRT except for Y-90 therapy) and control (no pre-LT LRT) groups. Of the patients who received pre-LT LRT, imaging studies were reviewed by two abdominal radiologists using 2018 LI-RADS criteria. Statistical analysis was performed using Kaplan-Meier survival curves and Cox proportional hazard models to assess OS and DFS.

**Results:**

No statistically significant difference in OS or DFS (*p* = 0.23 and *p* = 0.22 respectively) was initially found. Given significant difference in age between the groups (*p* < 0.0001), Cox proportional hazard models were used to adjust for age with statistical significance reached for better OS and DFS in the treatment group (*p* = 0.05 and *p* = 0.05 respectively). Contrary to our hypothesis, there was no difference between treatment response groups regarding overall survival or disease-free survival, presumably because of low number of HCC recurrences in our patient population (4%).

**Conclusion:**

Despite not reaching statistical significance, LI-RADS TR categorization demonstrates a good interreader agreement (Kappa 0.6), helping radiologists feel comfortable that modest sensitivity of the LI-RADS TR treatment response category for detecting pathologically active malignancy does not confer a negative clinical outcome.

## Introduction

Hepatocellular carcinoma (HCC) is projected to be the third most common cause of cancer death in United States, and its incidence is rapidly rising [1, 2]. Literature review forecasts anticipate a 137% rise in the incidence of HCC from 2015 to 2030 [3]. Metabolic associated fatty liver disease (MAFLD) and metabolic associated hepatic steatosis (MASH) are thought to play an important role in this trend, as continued increase in the rate of diabetes and metabolic syndrome due to genetic and environmental factors predispose to these disease states [4]. MAFLD and MASH both increase the risk of cirrhosis on their own and when combined with underlying viral hepatitis and alcohol liver disease, which in turn increase the risk of HCC [5]. These epidemiologic trends suggest that HCC will become an increasing burden on both individual patients and the healthcare system in the short-term future.

Liver transplant (LT) is a curative therapy for HCC within selected patients such as patients within Milan Criteria (MC). LT is effective because it removes malignant tissue from the body, while also addressing underlying cirrhosis which places patients at risk for HCC. Appropriately selected patients expect five-year survival rates as high as 70% after LT [6]. Patient selection for LT is of utmost clinical concern, as the supply of donor livers is insufficient to provide LT in all indicated cases [7]. The Milan Criteria (MC) were established in 1996 to help select patients with HCC who will have optimal outcomes after LT [8]. The MC suggest that optimal outcomes are obtained when HCC patients undergoing LT have one lesion less than or equal to 5 cm in diameter or up to three lesions less than or equal to 3 cm. In addition, patients should not have imaging evidence of vascular invasion or extrahepatic metastatic disease. Patients within MC have superior outcomes after LT compared to patients outside MC [9].

Locoregional therapy (LRT) can be used as bridging therapy to LT to help keep patients within MC and ensure their position on the waiting list and to actively downstage patients while being listed or considered for LT. Patients initially within MC on the waiting list for LT have as high as a 30% risk of falling off due to disease progression [8], and LRT can reduce that risk significantly by inducing necrosis in malignant cells. Bridging therapy is recommended for patients expected to be on the transplant waiting list for longer than 6 months [10]. Patients who are downstaged into MC prior to LT have similar 10-year survival rates to patients who always remained within MC before liver transplant [11], further underscoring the importance of LRT in the care paradigm for patients with HCC.

Various types of LRT can be used as bridging therapy, such as microwave or radiofrequency ablation (MWA/RFA), conventional or drug-eluting bead transarterial chemoembolization (cTACE/DEB-TACE), and Yttrium-90 transarterial radioembolization (Y-90 TARE). MWA/RFA involves directing a high energy probe under imaging guidance to the patient’s lesion of clinical concern. High energy microwave or radiofrequency energy is then delivered to the tumor, directly inducing coagulation necrosis of malignant cells. cTACE and DEB-TACE are superselective microcatheter directed therapies where the vascular supply of the tumor is identified under angiography. Various chemoembolic agents can then be delivered directly to the tumor through a microcatheter, where the combination of embolic and chemotherapeutic action induces cell death. Y-90 TARE is another superselective microcatheter based therapy which involves the delivery of radioactive Y-90 embospheres rather than traditional chemoembolic agents, which can result in a longer and more complete necrosis of the tumor cells [12]. MWA/RFA, cTACE/DEB-TACE, and Y-90 TARE can be used independently or in tandem, depending on the specific clinical scenario. These various locoregional therapies help keep patient within MC and thereby extend their eligibility to receive liver transplant [13].

The Liver Imaging Reporting and Data System Treatment Response Algorithm (LI-RADS TRA) is used to radiologically classify the response of HCC to LRT. LI-RADS TR observations can be described as LR-TR Nonviable, LR-TR Equivocal, LR-TR Viable or LR-TR Nonevaluable. According to the 2018 LI-RADS criteria & LI-RADS CT/MR Non-radiation v2024 algorithms [14, 15], LR-TR Nonviable lesions will demonstrate no arterial phase enhancement and will demonstrate an expected post treatment appearance. LR-TR Equivocal lesions will demonstrate an enhancement pattern that is not typical for expected treatment response, but they will not meet criteria for LR-TR Viable lesions. LR-TR Viable lesions demonstrate tissue that is nodular, mass-like or thickened along the treatment margin. In addition, LR-TR Viable lesions will demonstrate either arterial phase hyperenhancement, washout, or an enhancement pattern similar to pre-treatment imaging. LR-TR Nonevaluable category includes lesions which cannot be assigned to any of the previous categories due to degraded image quality, lack of IV contrast or failure to include the lesion of concern within the field of view of the exam. These lesions are usually followed by a repeat exam or short-term imaging follow up.

The LI-RADS TR algorithm has good specificity when assessing for incomplete treatment after LRT, however its sensitivity is more limited [16]. For example, LI-RADS TR may detect pathologically evident residual tumor after MWA/RFA with a sensitivity of only 40–77% [17]. For cTACE sensitivity is also low, with some studies demonstrating sensitivity of around 40% [18].

Patients with LR-TR Nonviable or LR-TR Equivocal lesions may therefore go on to have evidence of active malignancy on explant pathology evaluation. In other words, a liver that is radiologically negative for residual HCC after LRT has a reasonable probability of being positive for active disease upon pathologic examination [19]. A recent study by Hassan et al. on residual HCC after LRT found very low negative predicative value in excluding HCC at explant pathology, with approximately 75–77% of patients deemed LR TR Equivocal demonstrating residual tumors at explant pathology [20].

From literature we know that absence of viable HCC in native liver is an independent protective factor of tumor recurrence after liver transplantation. At present, it is unclear what impact the potential discordance between LR-TR classification and pathologic explant evaluation has on clinical outcomes such as overall survival (OS) or disease-free survival (DFS). In this retrospective review of 168 LT patients with pathologically proven HCC at explant evaluation, we aim to examine this important question. We hypothesize that modest sensitivity of the LI-RADS TR treatment response category for detecting pathologically active malignancy does not confer a negative clinical outcome. In fact, we hypothesize that the LI-RADS TR response category is associated with increased long-term survival and overall disease-free progression when compared to explant pathology. We also hypothesize that neoadjuvant LRT can improve OS and DFS in LT patients initially within MC.

## Materials/methods

**Fig. 1:**
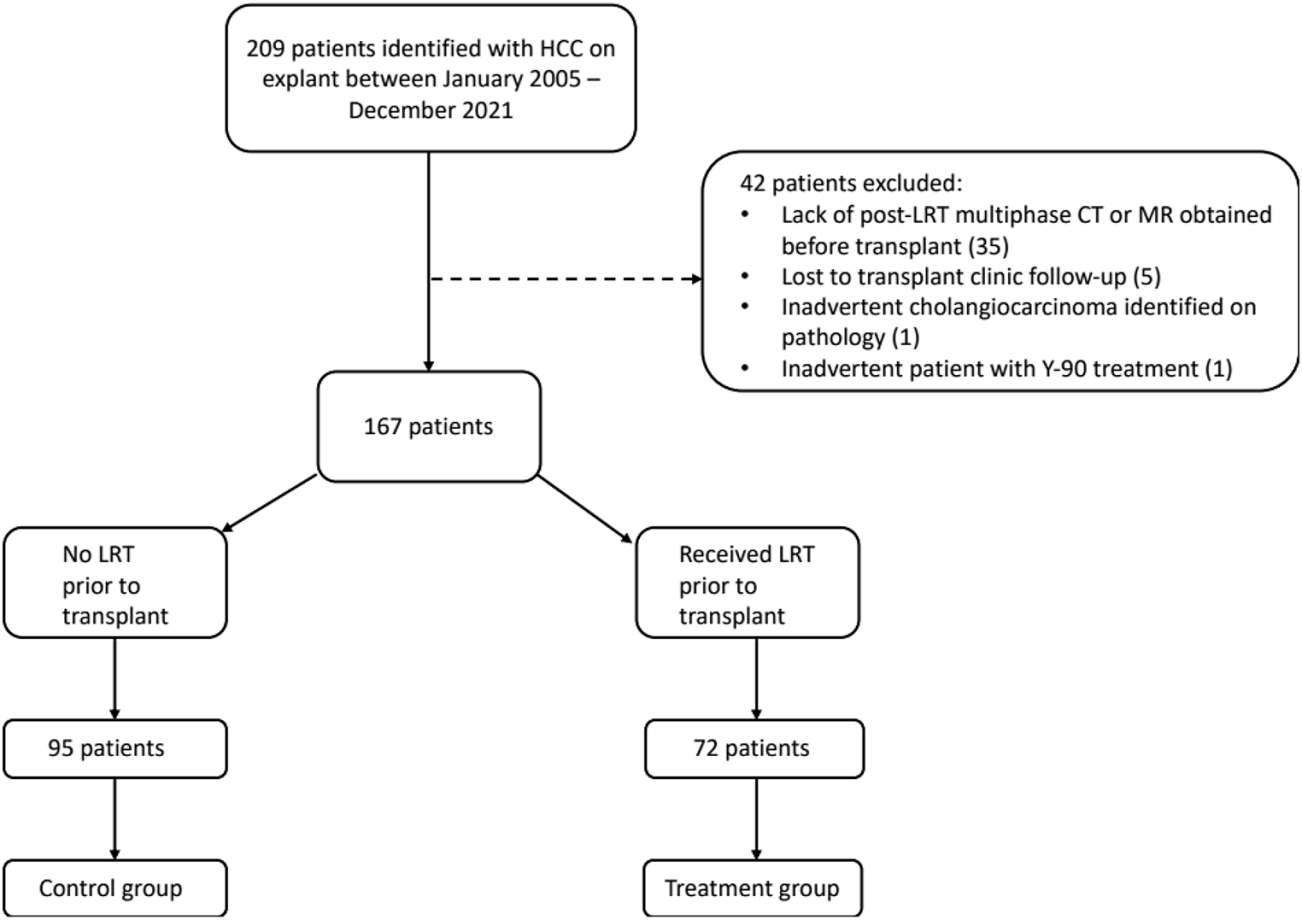
Fishbone diagram with exclusion criteria and patient distribution in control and treatment groups

A retrospective institutional review board (IRB) approved review was performed at our institution for patients who underwent liver transplant (LT) and were found to have hepatocellular carcinoma (HCC) on explant between January 2005 and December 2021. A total of 209 patients were initially identified with HCC on explant and exclusion criteria was applied: lack of post-treatment multiphase CT or MR obtained before LT, lost to transplant clinic follow-up, inadvertent cholangiocarcinoma found on pathology and Y-90/Radiation based therapies. Although Y-90 TARE is a more recent and popular therapy and given use of radiation spheres in this therapy, treatment assessment of residual tumor is confounded by ongoing slow necrosis of tumor and adjacent normal parenchyma. Therefore, we excluded patients with prior Y-90 TARE from our study cohorts, even if they had other non-radiation locoregional therapies (LRT) in the past. Therefore, 42 patients were excluded, resulting in a final cohort of 167 patients (Fig. 1). Clinical and imaging data, including age, gender, type of most recent locoregional treatment, date of last cross-sectional imaging before LT, LT date, and follow up data including mortality, cause of death, and date of HCC recurrence were collected. Demographic characteristics were summarized using counts and percentages for categorical variables and means and standard deviations for quantitative variables (Table 1). Of the patients who received pre-LT locoregional therapy, imaging studies were reviewed by two abdominal radiologists and 2018 LI-RADS treatment response categories were assigned accordingly. The original, clinical radiology reports were not considered in our analysis. Discrepant interpretations were adjudicated by a third abdominal radiologist.

**Table 1:** Patient demographics for the entire patient population (*n* = 167)

Variable	Sublevel	Summary Stats
Gender	Female	42 (25%)
	Male	125 (75%)
Age (years)		58.0 +/- 7.1 (range 32–70)
Recurrence	Yes	7 (4%)
	No	146 (87%)
	Unknown	14 (8%)
Mortality	Yes	72 (43%)
	No	91 (54%)
	Unknown	4 (2%)

### Statistical analysis

Differences in demographics characteristics between the control group (patients without pre-LT locoregional therapy) and the pre-LT locoregional treatment group were assessed using chi-square, Fisher’s exact, or two-sample t-tests, as appropriate. Inter-reader agreement in pre-LT LI-RADS treatment response was analyzed using Cohen’s kappa. Survival data were analyzed using Kaplan-Meier curves with differences between the control group and the pre-LT treatment group assessed using a log-rank test. Additionally, Cox proportional hazards regression models were used to analyze the association between overall survival and disease-free survival time after transplant with the presence or absence of pre-LT locoregional treatment, adjusting for patient age. In all cases, model assumptions were assessed using a combination of visual plots and formal testing.

Across all analyses, a p-value less than 0.05 was considered statistically significant. All analyses were performed using R, version 4.3.0 (R Foundation for Statistical Computing; Vienna, Austria) and JMP, version 17 (JMP Statistical Discovery LLC; Cary, NC).

## Results

The final cohort consisted of 167 patients, average age 58.0 years, 42 females (25%), and 125 males (75%) (Table 1).

A total of 95 patient underwent liver transplant without LRT (Control group), and 72 patients underwent liver transplant after LRT (Treatment group). Patients in the control group were younger than those in the treatment group (average age 55.9 and 60.8 respectively *p* < 0.0001). Length of follow up was: 5.1 +/- 2.9 years [range 0.0 to 10.6 years] for the treatment group, 7.3 +/- 5.5 years [range 0.0 to 17.7 years] for the control group and overall 6.4 +/- 4.7 years [range 0.0 to17.7 years]. The time interval between LRT and LT was 0.46 +/- 0.28 years [range 0.12 to 1.28 years]. There were 7 patients who developed recurrence after transplant for an overall recurrence rate of 4%, which is lower than that reported in the literature [11]. 6 recurrences were detected in the control group and only 1 recurrence was detected in the treatment group out of 72 patients (*p* = 0.13). Overall mortality was 53% (*n* = 50) in the control group and 31% (*n* = 22) in the treatment group (*p* = 0.002) (Table 2).

**Table 2:** Patient demographics for control and treatment groups with statistical analysis

Variable	Sublevel	Control (*n* = 95)	Treatment (*n* = 72)	*p*-value
Gender	Female	22 (23%)	20 (28%)	0.496
	Male	73 (77%)	52 (72%)	
Age (years)		55.9 +/- 7.4	60.8 +/- 5.4	< 0.0001
Recurrence	Yes	6 (6%)	1 (1%)	0.129
	No	78 (82%)	68 (94%)	
	Unknown	11 (12%)	3 (4%)	
Mortality	Yes	50 (53%)	22 (31%)	0.002
	No	41 (43%)	50 (69%)	
	Unknown	4 (4%)	----	

**Fig. 2:**
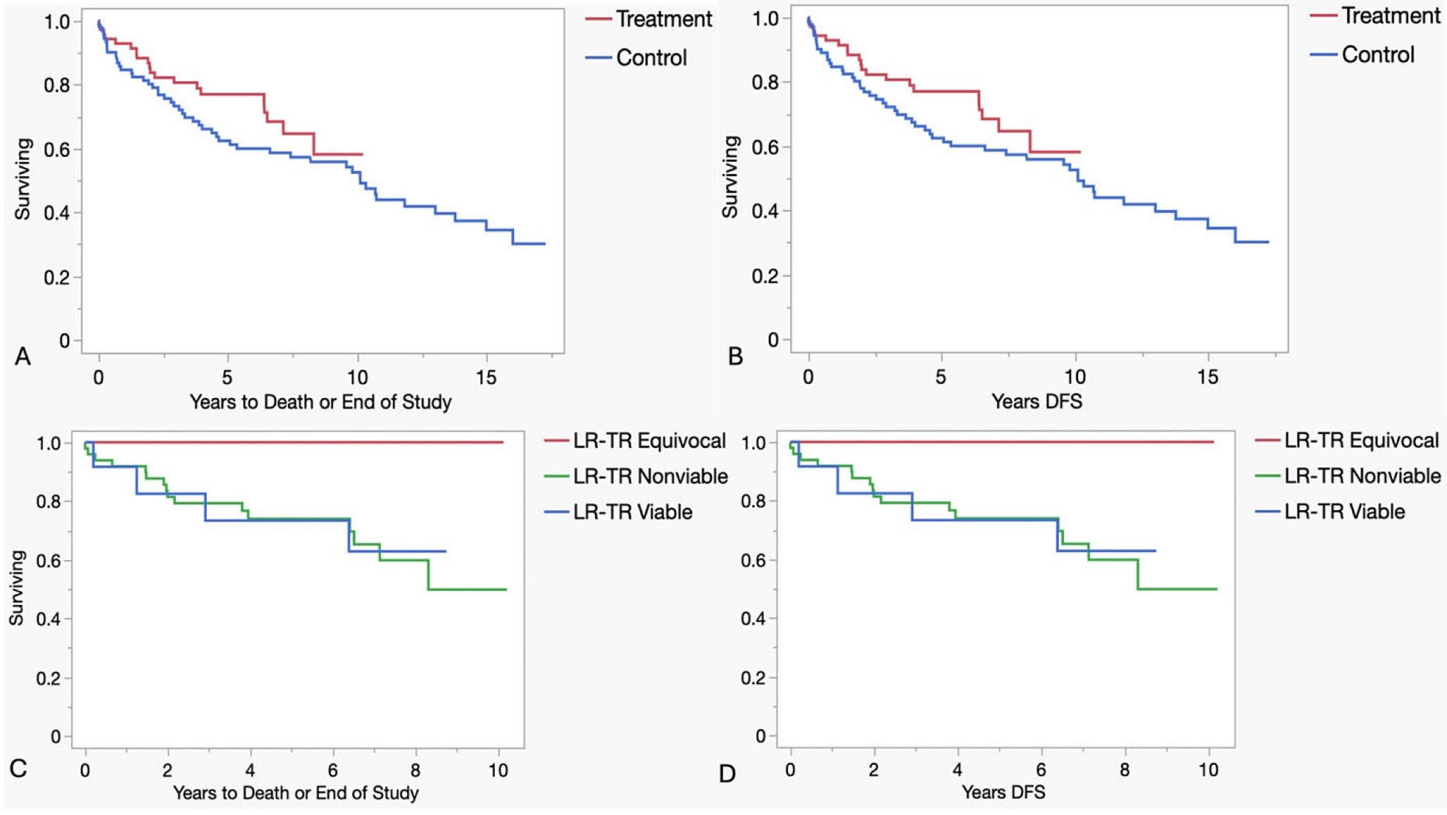
(**A**) Overall survival and (**B**) Disease-Free Survival (DFS) for treatment vs. control groups. (**C**) Overall survival and (**D**) Disease-Free Survival (DFS) for different treatment group LR-TR categories

Median overall survival (OS) and disease-free survival (DFS) for patients was 10.7 years. Log rank test demonstrated no statistically significant difference in OS or DFS (*p* = 0.23 and *p* = 0.22 respectively), between the control and treatment groups (Fig. 2; Table 3). Due to the significant difference in age between the groups, Cox proportional hazard models were used to adjust for age, and patients who received pre-transplant LRT had better OS and DFS (*p* = 0.05 and *p* = 0.05 respectively) compared to the control group. Cox model based and age adjusted treatment estimated hazard ratio were 0.58 (95% CI: 0.33 to 1.02) for both OS and DFS.

**Table 3:** Non-age adjusted OS and DFS values for control and treatment groups

**Group**	**Median OS (years)**	**Study Death Rate**	**5-Year OS**	**10-Year OS**
Control	10.1 years (95% CI: 6.6 to 15.0)	55%	62% (95% CI: 53–73%)	53% (95% CI: 43–65%)
Treatment	Not reached	29%	77% (95% CI: 67–88%)	58% (95% CI: 43–79%)
**Group**	**Median DFS (years)**	**Study Recurrence or Death Rate**	**5-Year DFS**	**10-Year DFS**
Control	10.1 years (95% CI: 6.6 to 15.0)	55%	63% (95% CI: 53–73%)	53% (95% CI: 43–65%)
Treatment	Not reached	29%	77% (95% CI: 67–88%)	58% (95% CI: 43–79%)

The treatment group underwent TACE (36), MWA (32), RFA (3), and bland embolization locoregional therapies (1) (Table 4). Overall, 94 lesions in 72 patients were rated and inter-reader agreement on a per lesion basis was fair (Kappa 0.40) and on a per patient basis was good (kappa of 0.6). Twelve patients were rated to have viable tumor (LR-TR viable 17%), 9 patients were equivocal for viable tumor (LR-TR equivocal 12%), and 51 patients were rated to have no evidence of viable tumor (LR-TR non-viable 51%). (Table 5) There was no difference between treatment response groups regarding overall survival or disease-free survival (*p* = 0.23 and *p* = 0.22 respectively) (Fig. 2). The only recurrence in the LIRADS-TR group occurred in a patient rated LR-TR-viable, but this difference did not achieve statistical significance (*p* = 0.30) (Table 6).

**Table 4:** Variation of different locoregional therapies (LRT) in the treatment group (*n* = 72)

Variable	Sublevel	Summary Stats
Last Localized Pretxp Type	TACE	36 (50%)
	MWA	32 (44%)
	RFA	3 (4%)
	Bland Embo	1 (1%)

**Table 5:** Variation of different LR-TR categories in the treatment group (*n* = 72)

Variable	Sublevel	Summary Stats
Final LR-TR Status	Viable	12 (17%)
	Equivocal	9 (12%)
	Nonviable	51 (71%)

**Table 6:** Recurrence rates in different LR-TR categories in the treatment group (*n* = 72)

Variable	Sublevel	Viable (*n* = 12)	Equivocal (*n* = 9)	Nonviable (*n* = 51)	*p*-value
Recurrence	Yes	1 (8%)	0 (0%)	0 (0%)	0.30
	No	11 (92%)	9 (100%)	48 (94%)	
	Unknown	0 (0%)	0 (0%)	3 (6%)	

## Discussion

Our initial hypothesis that LI-RADS TR response category has a closer correlation to OS & DFS than explant pathology did not hold true, most likely due to the very low recurrence rate of HCC in our study population. However, our results suggest that patients undergoing neoadjuvant LRT prior to LT have improved OS and DFS when adjusted for age. Inter-reader variability for LI-RADS TR response category was good on per-patient basis, these results with LI-RADS 2018 treatment algorithm can reassure radiologists that the modest sensitivity of the LI-RADS TR treatment response category in detecting pathologically active malignancy does not lead to a negative clinical outcome.

Several previous works have assessed the impact of bridging LRT on patient outcomes. A meta-analysis by Kostakis et al. included 26 retrospective studies examining the impact of neoadjuvant LRT on post-transplant outcomes [21]. A total of 9068 patients were included in the meta-analysis, with 6435 (71%) receiving neoadjuvant LRT. Based on this analysis, patients within Milan Criteria (MC) had improved 1-year survival after LT (95% CI 0.35–0.86). Improved survival status-post LRT may be due to induced necrosis of tumor cells, which can alter the local tumor microenvironment and modulate immune responses [22, 23] in addition to reducing the risk of local or distant metastasis. There was heterogeneity in the type of LRT included in the study, with patients undergoing MWA, RFA, TACE, and TARE. Only one study within the meta-analysis (Agopian et al. [24]) expressly included patients undergoing TARE within their cohort. Of note, this meta-analysis was somewhat limited in its analysis of radiologic response to LRT, as only 3 out of the 26 studies reported this data. In our study, patients who received pre-LT LRT had a lower mortality rate compared to those who did not. Although Y-90 TARE is increasingly utilized recently, the post treatment assessment of residual tumor is complicated by the ongoing slow necrosis of both the tumor and surrounding normal tissue. Therefore, we excluded those patients as recent guidelines recommend a two-tiered approach and that generalized LI-RADS criteria (for example LI-RADS 2018 criteria) should not applied for patients undergoing TARE or other radiation therapies.

**Fig. 3:**
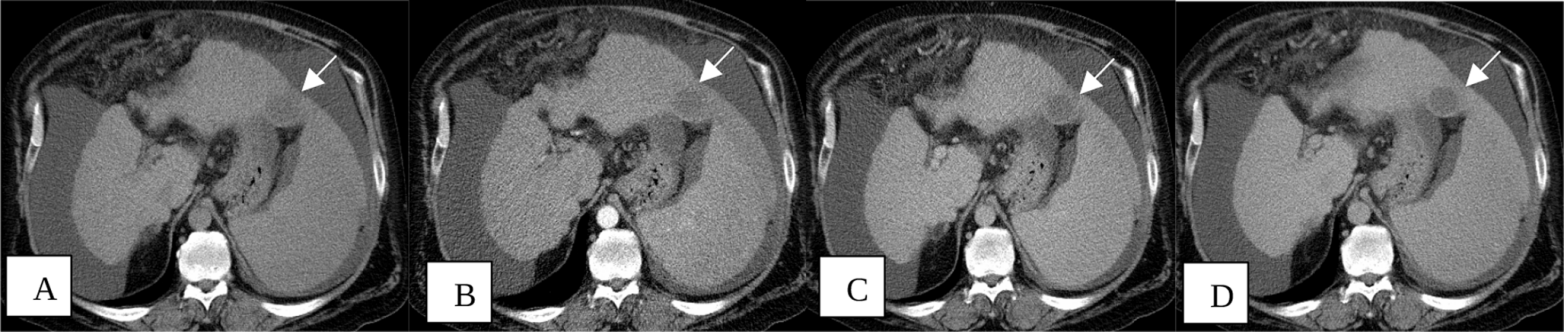
56-year-old male with cirrhosis, status post TACE for a 3.6 cm LI RADS 5 observation in segment III. Axial CT images before (**A**) and after intravenous contrast in the arterial (**B**), portal venous (**B**) and 3 min delayed (**C**) phases demonstrating a non-enhancing treatment cavity (white arrows) in keeping with LR-TR non-viable

**Fig. 4:**
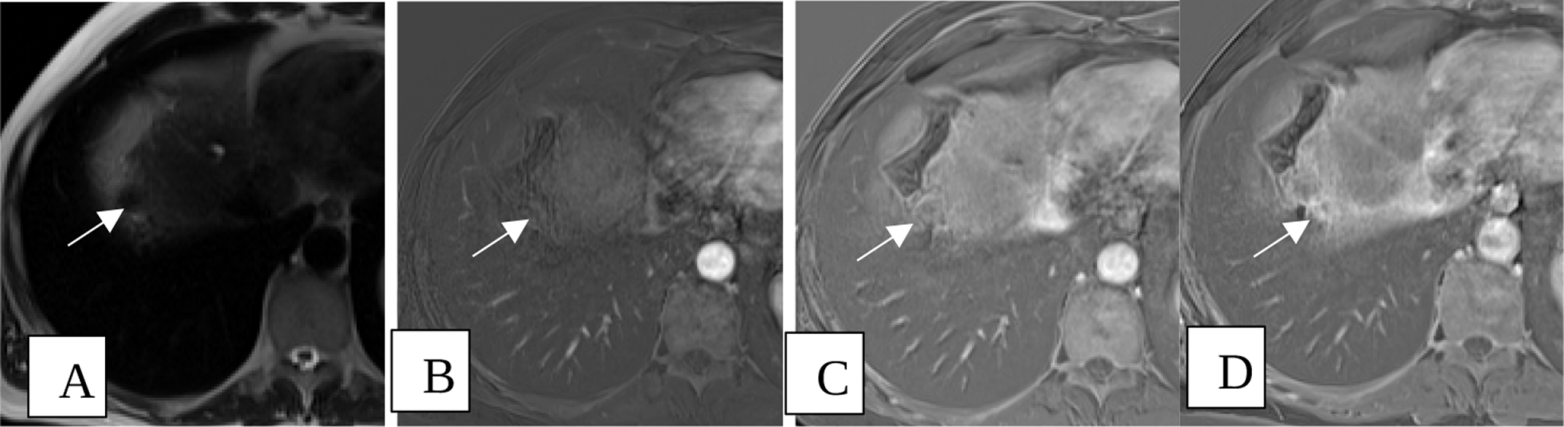
55-year-old male with cirrhosis, status post TACE for a 2.3 cm LI RADS 5 observation in segment VIII. Multiphasic MRI of the abdomen with axial T2 weighted images demonstrating a T2 hypointense lesion at the right hepatic dome (**A**). T1 weighted images were obtained before and after intravenous gadolinium contrast with subtractions performed in arterial (**B**), venous (**C**) and 5 min delayed phases (**D**) demonstrating increasing internal contrast enhancement within the cavity (arrows) in keeping with LR-TR equivocal

**Fig. 5:**
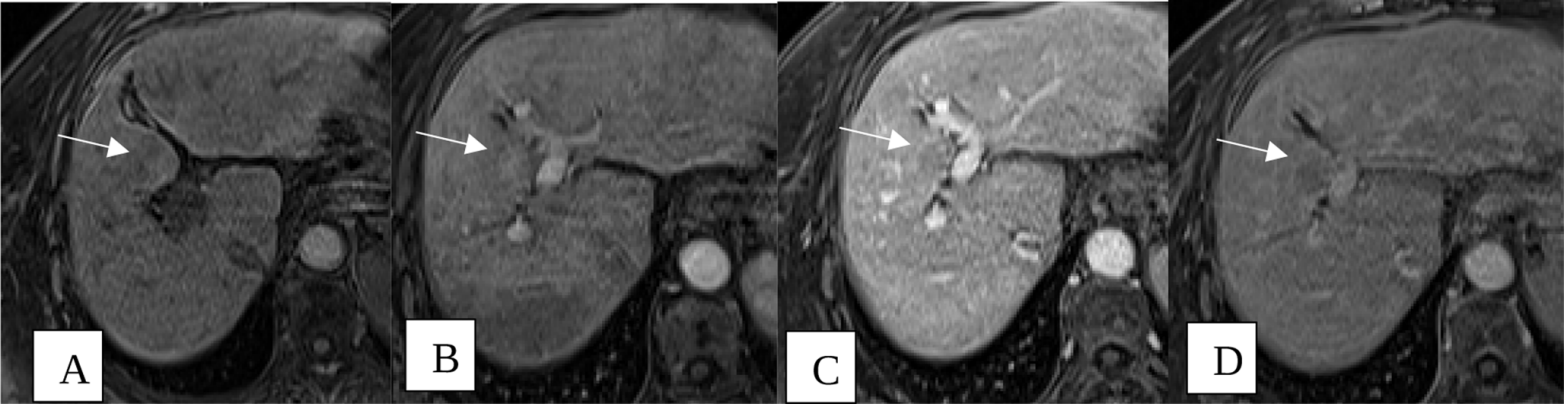
57-year-old male with cirrhosis status post TACE of 1.5 cm segment V/VIII LR-5 observation. Multiphasic MRI of the abdomen with pre-contrast T1 weighted image (**A**) demonstrating an ill-defined treatment cavity in segment V/VIII. After IV gadolinium contrast administration, there is heterogeneous enhancement in the arterial phase (**B**), with progressive washout on portal venous (**C**), and delayed 5-minute (**D**) T1 weighted images, consistent with LR-TR Viable

Radiologic response is a critical part of our current study. Treated lesions were predominantly TR-nonviable (Fig. 3), with 51 out of 72 patients (71%) having nonviable lesions. Nine patients (12%) had at least 1 TR-equivocal lesion (Fig. 4) and no TR-viable lesions. 12 patients (17%) were found to had TR-viable lesions (Fig. 5). Contrary to our hypothesis, there was no difference between treatment response groups regarding overall survival or disease-free survival (Fig. 2), presumably because of low number of HCC recurrences in our patient population (4%).

Our study has several strengths compared to the existing literature. First, our method of selecting patients based on explant pathology ensured that we had the largest possible sample of transplant patients who did in fact have HCC at our institution. This maximized our ability to isolate the treatment effect from neoadjuvant LRT. Second, good inter-reader variability existed between the patients included in our study. Although LI-RADS TR allows for more nuance than a schema such as mRECIST, which only considers arterial phase hyperenhancement in the determination of viability [25], there is still good interreader variability in our study. A kappa of 0.60 on per-patient basis implies that LI-RADS TR categories can have good integration into clinical practice. Third, the average follow-up time available for each patient was relatively long, with a patient cohort that extends dating back to 2005. Lastly, active exclusion of the radiation based locoregional therapies in our study even when LI-RADS TRA v2018 did not differentiate between radiation and non-radiation based therapies is a subliminal strength as the recent literature proves that similar treatment assessment criteria cannot be used interchangeably between the radiation and non-radiation based therapies. In particular, SBRT was found in certain studies to demonstrate persistent APHE even after successful treatment [26]. TARE can also demonstrate nodular mass-like enhancement after successful treatment [27]. The updated 2024 LI-RADS TR algorithm allows for these differences in treatment technique and describes the expected post-procedural appearance for both SBRT and TARE in the early (< 3 months) and late (> 6 months) post-radiation period.

Our study has several limitations. First, there was a relatively small cohort receiving pre-transplant LRT, with only 72 patients included. Second, there was an inability to determine if the lesions identified on explant pathology correlated with those seen on imaging, which could cloud our interpretation of imaging response. Third, there was a very low HCC recurrence rate in the study period, which may limit our ability to detect subtle differences in treatment groups. Only 7 out of 167 patients in the entire cohort (4%) and 1 out of 72 patients in the treatment cohort (1%) had HCC recurrence during the study. This is much lower than reported in the literature where recurrence rates of 13, 21 & 41% have been found for patients within MC, patients who were downstaged and patients who were not downstaged before the transplant respectively [11]. Fourth, there is some heterogeneity in our data as several types of LRT examined were within our patient cohort. Above heterogeneity can be explained by using data from 2005 to 2021 with advances in LRT occurring at a rapid rate, and practice patterns changing accordingly. Fifth, tumor characteristics and AFP level data was not calculated in this project. Sixth, a patient with even one viable lesion, was classified in the treatment group despite presence of other non-viable lesions. In other words, we did not assess outcomes per lesion rather per patient to better gauge the OS and DFS in our study. Lastly, our inter-reader agreement was not excellent as one would expect, which may be due to experience level of our abdominal radiologists and inherent subjectivity associated with LI-RADS 2018 criteria.

Future directions for this research could involve analyzing larger number of patients with HCC recurrence which would warrant multi-institutional studies and including a patient cohort with TARE therapies in the LRT arm. Some emerging evidence favors TARE over other forms of LRT for the treatment of HCC. For example, the TRACE randomized control trial demonstrated impressive improvements in time to tumor progression and median OS with TARE compared to DEB-TACE [28]. Although an older meta-analysis of five studies comparing TACE to TARE did not find significant treatment differences [29], these studies were limited by their retrospective design. It stands to reason that if further studies were to include a greater proportion of patients undergoing TARE therapy, there would be a more pronounced improvement in outcomes in the LRT group. LI-RADS Radiation and Non-radiation TRA v2024 will also be needed when analyzing treatment response for patients when including radiation-based therapies, which would make future studies more clinically relevant as radiologists have already transitioned to newer LI-RADS algorithms in their clinical practice at the time of this manuscript writing.

Although we did not find improved OS and DFS between different treatment groups (LR-TR viable vs. LR-TR equivocal vs. LR-TR Non-Viable), our study did find improved OS and DFS for patients undergoing neoadjuvant LRT prior to LT compared to control group when groups were adjusted for age. Age difference between the treatment and control groups was significantly different (*p* < 0.0001) and therefore adjusting for age was necessary in our study. With the above results and a good interreader agreement (Kappa 0.6) between different LI-RADS TR categories, this treatment algorithm can help radiologists feel comfortable that modest sensitivity of the LI-RADS TR treatment response category for detecting pathologically active malignancy does not confer a negative clinical outcome.

## Data Availability

No datasets were generated or analysed during the current study.
